# Induced plant responses to microbes and insects

**DOI:** 10.3389/fpls.2013.00475

**Published:** 2013-11-21

**Authors:** Corné M. J. Pieterse, Erik H. Poelman, Saskia C. M. Van Wees, Marcel Dicke

**Affiliations:** ^1^Plant-Microbe Interactions, Department of Biology, Faculty of Science, Utrecht UniversityUtrecht, Netherlands; ^2^Laboratory of Entomology, Department of Plant Science, Wageningen UniversityWageningen, Netherlands

**Keywords:** induced systemic resistance, systemic acquired resistance, insect-induced defense, priming, plant defense signaling, volatile organic compounds, microbiome, beneficial microbes

Plants play a key role in the sustainability of life on earth. They fix the solar energy that drives nearly all living processes. As a result, plants are members of complex communities and interact both with antagonists and beneficial organisms. To defend themselves against harmful pathogens and insects, plants have evolved a sophisticated immune system to perceive alien organisms, and to translate this perception into defense. The plant immune system is based on a surprisingly complex defense signaling network that is highly flexible in its capacity to recognize and respond to the invader encountered. Plant hormones and volatile organic compounds emerged as important signaling molecules in local and systemic induced defense responses to pathogen or insect attack.

Beneficial relationships between plants and microorganisms are frequent in nature as well, improving plant growth or helping the plant to overcome biotic or abiotic stress. Beneficial associations include root-colonizing microbes, such as plant growth-promoting rhizobacteria and fungi, and mycorrhizal fungi that form a symbiosis with ~80% of all plant species. These ecologically and agriculturally important beneficial associations are based on the exchange of resources between the plant and the mutualist. Each gram of soil contains billions of microbes. However, the microbial community on plant roots is very different from that in bulk soil. Hence, plants are able to recruit specific microbes from the soil to their roots. The establishment of beneficial associations requires mutual recognition and a high degree of coordination of plant and microbial responses through a continuous molecular dialog between the plant and the beneficial. Because beneficial microbes are recognized as alien organisms, active interference with the plant immune system is fundamental for the establishment of intimate mutualistic relationships.

An important question in plant defense signaling research is: how do plants integrate signals induced by pathogens, insect herbivores and beneficial microbes into an adaptive response that maximizes both profitable and protective functions? Molecular and genomic tools are now being used to uncover the complexity of the induced signaling networks that have evolved during the arms races between plants and the organisms with which they intimately interact. To understand the functioning of this complex signaling network in nature, molecular biologists and ecologists have joined forces to place molecular mechanisms of induced plant defenses in an ecological perspective. Exciting new discoveries have greatly advanced our understanding of how the co-evolutionary arms race between plants and its social network has shaped the plant immune system into a sophisticated defensive shield capable of warding off the majority of harmful organisms in its environment. Tremendous progress has also been made in the understanding of how plants respond to and benefit from mutualistic soil-borne microbes to maximize growth and survival. With 31 original contributions, this Research Topic provides a snapshot of the current state of the art of the field of induced plant responses to microbial pathogens, insect herbivores, and beneficial root-associated microbes, with a special focus on the translation of molecular mechanisms to ecology and vice versa.

In this Research Topic, a nice mix of Mini Reviews, Reviews, Original Research Articles, and Opinion Articles provide up-to-date information on diverse aspects of the plant immune system and how it functions against microbial pathogens. Newman et al. (2013) provide an update on the wide variety of microbe-associated molecular patterns (MAMPS) that to date have been characterized in bacterial, fungal and oomycetal pathogens. Locally triggered immune responses are often associated with systemic acquired resistance (SAR). This requires long-distance communication and signal amplification in distal plant parts. In recent years, many candidate molecules that function as systemically transported signals have been characterized (reviewed by Shah and Zeier, 2013). Other aspects of SAR, such as detailed insight in transcriptional reprogramming during SAR (Gruner et al., 2013), the role of the NPR1 regulatory NIMIN proteins in SAR (Hermann et al., 2013), and the effect of abiotic stress on the expression of SAR (Pye et al., 2013) are discussed in this Research Topic and provide interesting new insights into the field of SAR research. Plant hormones emerged as important regulators of local and systemic induced defense responses. Besides the classic defense-related hormones salicylic acid, jasmonic acid and ethylene, nitric oxide emerged as an important regulatory signal as well. In their Mini Review, Mur et al. (2013) integrate nitric oxide into the network of hormone-regulated immune responses.

More specific topics on plant-pathogen interactions are addressed by Zhang et al. (2013), who report on the role of the Sec61 ER protein transporting pore in susceptibility to powdery mildew. Singh and Zimmerli (2013) provide a nice review on the role of lectin receptor kinases in plant immunity. Yadeta and Thomma (2013) zoom in on the xylem as site of action when it comes to plant defense against vascular pathogens. Davidsson et al. (2013) provide insight into the state-of-the-art of pathogenicity of soft rot pectobacteria and the defensive machinery of plants to protect themselves against this group of pathogens. Cereal pathogens have major impacts on future food security. Ballini et al. (2013) and Balmer et al. (2013) describe how modern technology such as genetical genomics and metabolomics can help to investigate these scientifically and societally challenging host-pathogen interactions. On a more applied note, Abdul Latif et al. (2013) and Reglinski et al. (2013) describe how modeling approaches and fundamental knowledge on induced resistance can be used to develop control strategies in practice, such as to fight bacterial canker of kiwifruit.

A major topic in plant-insect interactions is the aspect of above-belowground interactions between plants, insects and other organisms. Wondafrash et al. (2013) review the field of systemic induced defense responses triggered by root parasitic nematodes and their effects against herbivorous insects on foliar tissues. In their original research articles, Paudel et al. (2013) and Schweizer et al. (2013) provide novel insights into the role of different transcription factors and the cellular redox status in the regulation of induced defense against caterpillar herbivory. Sap-sucking insects have a completely different mode of action. De Vos and Vandoorn (2013) review current knowledge on resistance to this group of insects in modern-day agriculture.

Plant volatiles emerged as important signals in the communication between plants, insect herbivores, and their enemies. In this Research Topic a number of contributions address the role of volatile organic compounds in the communication of the plant's social network. Von Mérey et al. (2013) describe the role of herbivore-induced plant volatiles in the attraction and feeding behavior of a caterpillar herbivore. Scala et al. (2013) investigated the effect of a common herbivore-induced plant volatile on a bacterial plant pathogen. Rodriguez-Saona et al. (2013) investigated the role of jasmonate-mediated induction of plant volatiles on multi-trophic level interactions in American cranberry, while Niinemets et al. (2013) addressed the question how volatile emission patterns induced by biotic stresses relate to the degree of damage. After release into the atmosphere, the plant no longer controls the action of its produced volatiles. In their review “Where do herbivore-induced plant volatiles go” Holopainen and Blande (2013) summarize the potential ecological and atmospheric processes that involve the reaction products of plant volatiles that are formed in the atmosphere upon release by the plant.

An emerging theme in the field of plant-microbe interactions is the importance of beneficial microbes in plant health. Bakker et al. (2013) reviewed the field of root microbiomics and highlights the role of root-associated beneficial microbes in plant growth and protection. Studies by Carvalhais et al. (2013), Hol et al. (2013) and Yi et al. (2013) provide additional insight into the roles of plant growth-promoting rhizobacteria in plant health, either as stimulants of the plant's immune system or through optimizing the root microbial ecology. Paszkowski and Gutjahr (2013) provide an excellent review on the mechanisms by which arbuscular mycorrhizal fungi shape the architecture of plants roots, which is essential for optimal acquisition of mineral nutrients and water from the soil. Furthermore, novel insights into mechanisms by which plant growth-promoting fungi, such as *Piriformospora indica* and *Trichoderma* spp., interact with the plant immune system or antagonize pathogens in the soil are provided by contributions of Martinez-Medina et al. (2013), Rafiqi et al. (2013) and Studholme et al. (2013).

With this Research Topic we aimed to provide a platform for scientists who liked to share their understanding of how induced plant responses shape the plant's social network. The excellent contributions are a demonstration of a highly active research community in plant science. Together they provide a detailed understanding of the intrinsic capacity of plants to simultaneously accommodate mutualists and ward off enemies to maximize both growth-stimulating and protective functions.
